# Septic Root Canals

**Published:** 1889-04

**Authors:** W. E. Tucker

**Affiliations:** Butler, Mo.


ARTICLE IV.

### SEPTIC ROOT CANALS.

DR. W. E. TUCKER, BUTLER, MO.

In teeth of this class, pulps have been dead long enough to become gangrenous, or for complete putrefaction to ensue, an abscess, either blind or with a fistulous opening, may have resulted; in either case the preliminary steps in the treatment are just the same. First remove the loose debris, and at least a portion of the decalcified dentine, and, if it is possible, the rubber dam should invariably be applied at this stage. After drying the cavity and saturating it with a disinfectant, cleanse it of decay before entering the pulp-chamber. The importance of suitable disinfectants now presents itself. There are quite a number, but I will only refer to a few. Peroxide of hydrogen is excellent and a great favorite with many, but as it cannot be obtained pure and fresh in many places, it may be dispensed with. Scientific investigation having demonstrated the fact that wherever fermentation and putrefaction exist there are present micro-organisms, it is clear that in the removal of septic matter the dentist needs something that will effectually destroy bacteria, and, if possible, prevent their reformation. I do not see the necessity of using a half-dozen different remedies if one or two will accomplish the desired result. For my part, I like to know which medicine to depend on, and, if I use different kinds in a given case, I would be like the old woman who objected to taking more than one kind of medicine, for in case she should be so fortunate as to get well, she would never know which cured her. For some time I have been using bichloride of mercury almost to the exclusion of anything else, and it has given great satisfaction. It is a very powerful germicide, even when the solution is so weak as not to injure the most delicate tissues of the mouth. To make a suitable solution, which is one part to a thousond, add to 1 ounce of distilled rain water, ½ grain of bichloride of mercury. As the bichloride of mercury decomposes or becomes precipitated, it should be made fresh quite frequently, possibly once a week. Some one has suggested the addition of four or five drops of muriatic acid to the ounce, claiming that it not only increases its power, but also prevents precipitation. Iodoform has been much used in the treatment of the teeth, but it cannot be depended on as a destroyer of microbes, tho it is excellent in preventing their reformation, and hence is useful in combination with chloro-percha for root-filling. Eugenol will be found quite useful as a disinfectant, antiseptic, deodorizer, and obtundent. It is not caustic, but very penetrating.

But to return to our subject of cleansing a tooth of septic matter: The cavity of decay being cleansed, the pulp-chamber should be thoroughly opened and enlarged, but without defacing the natural surface of the pulp-chamber near the entrance to the root-canals; wipe the cavity occasionally with cotton dipt in the bichloride solution. In small roots it is often necessary to enlarge the opening a little, that they may be more easily entered. Now, with a small broach and cotton dipt in the solution, enter the root canal only a short distance, rotate, and remove; wipe the cotton from the broach on tissue or soft white paper, never on a napkin. Take fresh cotton, going a little deeper into the canal each time, and continue the process till you have reached the apex and the canal is cleansed of all septic matter, and all odor of mephitic gas is destroyed. The cotton should now come from the tooth clean and white. Dry with cotton and hot air, and fill as above, immediately. If you cannot succeed in getting the canal perfectly dry, it is evidence of a blind abscess, and I would not fill immediately; but after flooding the canals with the solution, or eugenol, fill the cavity loosely with cotton, and wait a few days, at the close of which time, if the root can be made perfectly dry, the process may be completed without further delay. If peroxide of hydrogen is at hand, it may be used successfully in expelling the pus, by forcing it into the sac; then the root may be filled immediately. Of course, if the root is very sore and the flow of pus very considerable, immediate root-filling is not indicated. Free vent for the pus having been made, leave the cavity open a few days. If the abscess has a fistulous opening and the tooth is not very sore, after cleansing the canals, force the solution freely through the fistula, and fill immediately.

Now all this may seem easy to one just beginning the practice of dentistry. It is true, a large majority of cases are quite simple and easy, but many present themselves that will tax the patience and skill of those of long experience. Sometimes the mouth is small, and the tooth difficult of access; sometimes it seems almost impossible to apply the rubber dam; and sometimes it is difficult to find the entrance to the root-canals, and to cleanse and fill them after being found. Then it will require an extra effort to gain free access to the root, it sometimes being necessary to cut away a considerable portion of the crown. To apply the rubber dam, where the cavity runs below the gum it may be necessary to temporarily fill with gutta percha, being careful not to force it into the pulp-chamber, and then make a new opening; or in some cases, to put a gold band around the tooth, and then apply the dam. The short piano-wire reamers are, with me at least, indispensable in finding and reaming out those difficult root-canals. It is almost impossible for them to break, and while it is unsafe to drill into roots, they can with safety be reamed out a little, thereby making the entrance of the canals more easy. A pair of light pliers, such as those belonging to Howe's set of crowning instruments, are often useful in placing these little reamers, which are then to be rotated with the thumb and finger. Now the short broaches, wound with fibers of cotton, are to be used in these difficult canals, remembering that the rotary movement is necessary to remove the contents of a canal. If merely forced into the canal, it will act as a piston and force everything toward the end of the root, instead of entangling the disintegrated nerve tissue in the fibers of cotton.—*Western Journal.*

